# Invasive Trichosporonosis in Neonates and Pediatric Patients with Malignancies or Hematologic Disorders

**DOI:** 10.3390/pathogens11020242

**Published:** 2022-02-12

**Authors:** Maria Kourti, Emmanuel Roilides

**Affiliations:** Infectious Diseases Unit, 3rd Department of Pediatrics, School of Medicine, Faculty of Health Sciences, Aristotle University of Thessaloniki, Hippokration General Hospital, 54642 Thessaloniki, Greece; roilides@gmail.com

**Keywords:** *Trichosporon*, trichosporonosis, neonate, hematologic disorder, malignancy

## Abstract

(1) Background: *Trichosporon* species have emerged as important opportunistic fungal pathogens, with *Trichosporon asahii* being the leading and most frequent cause of invasive disease. (2) Methods: We performed a global review focused on invasive trichosporonosis in neonates and pediatric patients with malignancies or hematologic disorders. We reviewed case reports and case series of trichosporonosis due to *T. asahii* published since 1994, the year of the revised taxonomic classification. (3) Results: Twenty-four cases of invasive trichosporonosis were identified in neonates with the presence of central venous catheter and use of broad-spectrum antibiotics recognized as the main predisposing factors. Thirty-two cases were identified in children with malignancies or hematologic disorders, predominantly with severe neutropenia. *Trichosporon asahii* was isolated from blood in 24/32 (75%) pediatric cases. Cutaneous involvement was frequently observed in invasive trichosporonosis. Micafungin was the most commonly used prophylactic agent (9/22; 41%). Ten patients receiving prophylactic echinocandins were identified with breakthrough infections. A favorable outcome was reported in 12/16 (75%) pediatric patients receiving targeted monotherapy with voriconazole or combined with liposomal amphotericin B. Overall mortality in neonates and children with malignancy was 67% and 60%, respectively. (4) Conclusions: Voriconazole is advocated for the treatment of invasive trichosporonosis given the intrinsic resistance to echinocandins and poor susceptibility to polyenes.

## 1. Introduction

*Trichosporon* species are basidiomycetous yeast-like fungi, which are characterized by the formation of arthroconidia that disarticulate from septate hyaline hyphae [1]. The word *Trichosporon* is derived from Greek words Tricho (hair) and Sporon (spores). *Trichosporon* species are found in nature, soil, water, mammals, birds, bats and cattle and also colonize the human skin, gastrointestinal tract and mucosal surfaces as part of the human microbiota [2,3]. They are also responsible for superficial infections (white piedra), allergic pneumonitis and rarely invasive infection [4,5,6,7,8,9,10].

Since the first case of invasive *Trichosporon* infection (ITI) reported by Watson and Kallichurum in 1970, *Trichosporon* species have emerged as important opportunistic fungal pathogens [11]. *Trichosporon asahii,* in particular, is considered to be the leading and most frequent cause of invasive disease [12]. Invasive *Trichosporon* infection may involve many organs, while *Trichosporon* fungemia (TF), including catheter-related fungemia, represents the main type of this opportunistic infection, which accounts for between 58.8 and 74.7% of infections [13,14]. In the 1980s, Walsh et al. reported ITI as the second most common cause of fungemia in patients with hematological malignancies [15]. As triazole derivatives became widely available, the incidence of ITI decreased in early the 2000s [16] followed by a re-emergence of *Trichosporon* as an increasingly common pathogen in immunocompromised hosts after the wide use of echinocandins [16,17,18,19]. Most cases of invasive infection are seen in patients with neutropenia and malignancy, especially in adults and children with hematological malignancies and intravascular indwelling catheters. Premature neonates with a low birth weight, patients with Acquired Immune Deficiency Syndrome (AIDS) and critically ill patients exposed to broad-spectrum antibiotics are also at increased risk [20,21,22,23,24,25,26,27]. Data regarding ITIs in children are based on case reports and small case series. Therefore, we performed this global review in order to increase scientific knowledge and evaluate existing therapeutic strategies aiming for the optimal patient outcome.

## 2. Results

The literature review yielded 24 neonatal cases and 32 ITIs in children with malignancy or hematologic disorder that fulfilled our inclusion criteria. These cases constituted the basis of the present review (Table 1 and Table 2).

### 2.1. Neonates

Twenty-four cases of ITI due to *T. asahii* in neonates were identified. The female/male ratio was 1.14/1. Several occasional outbreaks of trichosporonemia from different Neonatal Intensive Care Units (NICUs) were reported [28,29,30,31,32,33,34,35,36,37,38]. Two outbreaks in a single NICU in India contributed to 11/24 (46%) cases [39,40]. In general, the geographic distribution involved three continents, while many cases originated from countries with temperate and subtropical climates. The median birth weight (BW) of neonates was 960 gr and the median gestational age (GA) was 27 weeks. Among 21 cases with data reported, 19 (90%) occurred in premature neonates. The median postnatal age at diagnosis was 11 days. The presence of central venous catheter (CVC) and use of broad-spectrum antibiotics were reported in the vast majority of cases. Fungemia was reported in 22/24 (92%) neonates. Other specimens that grew *Trichosporon* spp. were urine, tracheal aspirate and peritoneal fluid. Conventional amphotericin B (AMB) or liposomal amphotericin B (LAMB) were the most frequently used monotherapies. Voriconazole (VRC) exhibited the lowest median minimum inhibitory concentration, (ΜΙC) (0.03 μg/mL) value against *T. asahii*. Overall mortality in neonates was 16/24 (67%).

### 2.2. Malignant and/or Hematologic Disorders

Thirty-two cases of ITI were identified related to malignant and/or hematologic disorders. The most common underlying disorder was acute lymphoblastic leukemia (ALL) (13/32; 41%) followed by acute myeloid leukemia (AML) (8/32 cases; 25%). The remaining 34% of the reported cases included three cases with aplastic anemia, two cases with mixed ALL and one case with Blackfan–Diamond, myelodysplastic syndrome (MDS), Langerhans cell histiocytosis, Wilms tumor, Ewing sarcoma and yolk sac tumor, respectively [38,41,42,43,44,45,46,47,48,49,50,51,52,53,54,55,56,57,58,59]. The male/female ratio was 1/1. The median age was 11.5 years (range:1–18 years). Details about neutropenia were available in 23/32 (72%) patients and the majority (22/23; 96%) developed severe neutropenia (Absolute Neutrophil Count (ANC) ≤ 0.5 × 10^9^ neutrophils/L). *Trichosporon asahii* was isolated from various types of clinical specimens including blood in 24/32 (75%) patients. Cutaneous involvement with papulonodular or pustular lesions was common and was frequently observed in 7/32 (22%) patients. Susceptibilities of *T. asahii* to various antifungal agents are shown in Table 3. Of the 32 patients with *T. asahii* disseminated infection, 18 (56%) had an in vitro susceptibility test. Voriconazole exhibited the lowest median Minimal Inhibitory Concentration (MIC) (0.06 μg/mL) value against *T. asahii*.

Information about antifungal prophylaxis given before ITI was available in 18/32 (56%) patients, and micafungin was the most commonly agent used (9/18; 50%). Ten patients receiving prophylactic echinocandins (55%) were identified with breakthrough infections. Various empirical treatment regimens were used; LAMB was used as monotherapy in 8/20 (40%) patients and in combination with an azole in 2/20 patients (10%). As targeted monotherapy VRC was administered in 11/22 (50%) patients. The combination of an azole with LAMB was reported in 6/22 (27%), especially VRC in combination with LAMB in 5/22 (23%). A favorable outcome was reported in 12/16 (75%) in patients receiving VRC as monotherapy or in combination with LAMB. Overall mortality in pediatric patients with malignancy and ITI was 60%

## 3. Discussion

According to our review, invasive trichosporonosis is rarely documented in children and is mainly reported in premature neonates and in immunocompromised children with hematological malignancies. *T. asahii* is the predominant *Trichosporon* species that causes invasive infection, especially breakthrough infections in patients receiving prophylactic/empirical antifungal treatment [1,60,61]. It is noteworthy that all pediatric cases are reported in the second half of the 2000s, indicating the re-emergence of this opportunistic fungal pathogen. After candidiasis, trichosporonosis is considered the second most frequent yeast infection leading to fungemia in patients with hematological malignancies [62,63,64]. Moreover, a change in the geographical distribution of cases is noticeable in the second decade of 2000s, since more cases have been reported from South America and Asia. An increasing concern of physicians, as well as the wider availability of more sophisticated molecular diagnostic methods, have played a role but the real epidemiological trend remains to be established.

In pediatric cancer patients, the largest group is comprised of leukemia patients. Among them, patients with ALL are at lower risk for invasive fungal infections (IFIs) compared to children with leukemia relapse or AML. Nevertheless, ALL patients are the largest group in absolute numbers reported with IFIs in children [65]. Our literature review revealed a total of 32 children with malignant and/or hematologic diseases. Among children with hematological malignancies, the majority were children with ALL. In contrast, among adults, the majority were AML patients followed by ALL and MDS [62]. Profound and prolonged neutropenia is an established risk factor for IFIs. In accordance with this, when the ANC was reported, the vast majority of children had neutropenia, highlighting the importance of neutrophil recovery in the prevention of ITI. Moreover, the use of broad-spectrum antibiotics and concomitant bacteremia play a significant role in the imbalance of the microbiota, resulting in potential IFI. Prolonged and severe neutropenia, together with the underlying immune status of the host, play a critical role in the outcome of the infection in children with hematologic or malignant disorder and in neonates [66,67]. The presence of a CVC and the disruption of the mucosal barrier might provide a portal of entry for *Trichosporon* spp. The formation of *Trichosporon* biofilms on catheter surfaces is important in the pathogenesis of invasive trichosporonosis [68]. Therefore, catheter removal as source control should be suggested whenever feasible.

Diagnosis is challenging since it relies on the isolation of a yeast-like organism from a clinical specimen. Direct examination seldom contributes to a definite diagnosis as it rarely demonstrates arthroconidia and it resembles *Candida* in histology. However, it has thinner hyphae and pseudohyphae and is slightly stained with Gomori methenamine silver (GMS) stain. Cutaneous involvement with maculopapular or pustular lesions that are sometimes necrotic is suggestive of trichosporonosis, though it may also be present in disseminated candidiasis. Biopsy and culture specimens of cutaneous lesions are helpful in establishing the diagnosis. Galligan et al. reported a child with relapsed ALL and disseminated *T. asahii* infection that had cutaneous nodules suggestive of fungal infection [42]. Despite the fact that histologic characteristics resembled Neutrophil Eccrine Hydradenitis, staining with periodic acid-Schiff stain and GMS confirmed the diagnosis of trichosporonosis. Moreover, de Almeida et al. has shown that MALDI–TOF spectrometry could be used as a valuable alternative for routine identification [62]. Direct sequencing of the IGS1 region of the ribosomal DNA is considered the reference method for species identification of *Trichosporon* isolates [69]. The timing and sensitivity of the diagnostic method is an important factor for successful management of ITIs. Invasive trichosporonosis can involve many organs, but *Trichosporon* fungemia (TF) including catheter-related fungemia, represents the main type of this opportunistic infection, as depicted in this review.

Prompt initiation of proper antifungal therapy is considered critical for obtaining a favorable outcome. Global guidelines for the management of rare yeasts from the European Confederation of Medical Mycology in collaboration with the International Society for Human and Animal Mycology and American Society for Microbiology have recently been published [70]. Various antifungal agents are available in the treatment of invasive trichosporonosis.

For the neutropenic pediatric patients with potential IFI, prophylactic/empirical treatment with echinocandins or a formulation of AMB has been recommended. Review of the literature revealed ten pediatric cases with breakthrough infections in patients receiving prophylactic echinocandins. Echinocandins are ineffective against *Trichosporon* spp. Moreover, it has been reported that their use may select for resistant fungal organisms, which explains the re-emergence of this opportunistic fungal pathogen [71]. Amphotericin B has shown some positive effectiveness against *Trichosporon* spp. in vitro, but it functions poorly with breakthrough infections, particularly in patients with profound neutropenia on high doses of AMB [72]. Walsh et al. reported that 77% of *Trichosporon* isolates were not killed at achievable AMB serum levels and this finding was correlated with refractory, disseminated trichosporonosis in neutropenic patients [73]. Poor response to AMB has also been reported in adult patients [1,13,67,74]. Nevertheless, successful results with AMB have been reported in neonatal cases with disseminated disease [30,37,39]. Variable susceptibility to AMB in vitro and in vivo may be explained by the production of a biofilm layer. The capability of *T. asahii* to produce biofilms is well documented in vitro [68]. In addition, an increased antifungal resistance to AMB has been reported to be directly proportional to increased biofilm production [75]. Therefore, the expected response to AMB may not be observed in the clinical setting, despite the in vitro sensitivity to AMB. Resistance to AMB and echinocandins is alarming not only for pediatric patients with neutropenia but also for neonates since they are both commonly used as systemic antifungal agents in preterm neonates. Early diagnosis of trichosporonosis remains a challenge since *Trichosporon* spp. may be less susceptible to empirical or prophylactic antifungal drugs that are frequently used, such as echinocandins and AMB. *Trichosporon* spp. seem to be sensitive to AMB in vitro, but this response may not be observed in vivo when a biofilm layer is produced by *Trichosporon* spp.

Although in vitro and in vivo studies have shown that *Trichosporon* species are resistant to the fungicidal effect of AMB, antifungal triazoles have been found to be fungicidal against *Trichosporon* species [67,73]. A favorable outcome in patients who received a VCZ regimen or an AMB–triazole combined regimen was reported by Liao et al. who assessed 185 cases of *Trichosporon* fungemia [76]. Moreover, Almeida et al. reported that azole-based therapy was a protective factor against adverse outcomes in 199 cases of proven infection and in 4 cases of probable infection caused by *Trichosporon* spp [62]. In accordance with this, a favorable outcome was reported in 12/16 (75%) pediatric patients receiving targeted monotherapy with VRC or combined with LAMB. However, fatal pediatric cases have been reported in children despite treatment with AMB and VRC [53].

Considering the intrinsic resistance to echinocandins and poor susceptibility to polyenes, triazoles have been proposed as the antifungals of choice for invasive trichosporonosis [77]. Azole-including therapy was more frequently used especially after 2004 [14]. Global guidelines published in 2021 moderately recommend VRC as the initial antifungal therapy, whereas fluconazole is also moderately supported, contingent on the MIC. Weak support exists for combination antifungal therapy [70]. The first successful treatment of ITI with voriconazole was reported in 2002 [78], a finding that has been confirmed in adults and children with IT [79]. According to our results, targeted monotherapy with VRC was reported in 11 pediatric patients with malignancy or hematologic disorder, resulting in a favorable outcome in 8/11(73%) patients. Combination therapy with AMB and a triazole has not been proven to be superior to VRC alone in vitro and requires more clinical studies to be confirmed [1,62,80]. Our review suggests that azole-including therapy may be superior to echinocandin- or AMB- based therapy in children, as it is in adults. According to the recently published global guidelines for the management of rare yeast infections, azole-polyene combinations should be reserved for salvage therapy [70]. Nevertheless, lately multi-drug resistant *Trichosporon* spp. has been reported with the increased use of broad-spectrum triazoles for prophylaxis in high-risk patients [81]. Multiple drug interactions in patients receiving chemotherapy and pharmacokinetic variability may play a role in the subtherapeutic level of triazoles leading to resistance to triazoles.

## 4. Materials and Methods

Published cases and case series of ITI in neonates and patients aged ≤18 years with malignancy or non-malignant hematologic disorder were reviewed. Only original full-text articles were included in the analysis. We searched PubMed for publications of case series and single case reports of ITI with the following keywords: “Trichosporon”, “trichosporonosis”, “invasive infection”, “neonates”, “child”, “pediatric malignancy”, “leukemia”, “tumor”. Moreover, the reference list of each article was further assessed in order to verify that all published cases were included in this review. This search was conducted between 1994 (the year of the revised taxonomic classification) and December 2020. Since the vast majority of ITI is due to *T. asahii and* isolates from invasive deep infections previously reported in the literature as *T. beigelii* and/or *Trichosporon cutaneum* would belong to *T. asahii*, we limited the search to *T. asahii*. Invasive trichosporonosis was defined according to the definitions of invasive fungal disease of the European Organization for Research and Treatment of Cancer/Invasive Fungal Infections Cooperative Group and the National Institute of Allergy and Infectious Diseases Mycoses Study Group consensus group (EORTC/MSG) [82]. Neutropenia was defined as an ANC ≤ 0.5 × 10^9^ neutrophils/L at the time of *Trichosporon* isolation. Invasive fungal disease was defined as proven infection according to the revised definitions by the EORTC/MSG consensus group [28]. Cases of superficial infection and infection with *Trichosporon capitatum* or *Trichosporon pullulans* were excluded as they have been reclassified to a different genus. A master Excel database was created containing study characteristics (first author name, year of publication, geographic location) demographic (sex, age, GA, and BW for neonates), underlying condition, microbiology data including antifungal susceptibility testing (AST), prophylactic/empiric or targeted antifungal treatment, and clinical outcome.

## 5. Conclusions

Trichosporonosis is an emerging concern in preterm neonates treated with broad-spectrum antimicrobials and indwelling catheters, and in children with hematologic malignant disease receiving prophylaxis or treatment with echinocandins given their lack of efficacy against this yeast. Treatment remains challenging due to the rarity and resistance to standard antifungals and the compromised status of the host. Invasive infection caused by *T. asahii* is a rare but potentially fatal complication of the immunosuppression associated with cancer treatment and immaturity of the immune system of younger children and should be considered in the differential diagnosis, especially in patients with neutropenia and recalcitrant fever. Voriconazole is considered as the first-choice therapy followed by fluconazole as an alternative. Combined therapy with an azole and LAMB is advocated as salvage therapy. Removal of the CVC and recovery of neutropenia are also considered as key factors for improved outcome. Prompt and aggressive treatment of ITI is important since *T. asahii* is less susceptible to the recommended empirical or prophylactic antifungal regimens.

## Figures and Tables

**Table 1:** Characteristics of neonatal invasive infections due to *Trichosporon* spp.

Author (yr)	Postnatal Day	Species	Sex	Site of Isolation	Weight (gr)	Gestation Age (wks)	Medical History	Treatment	Outcome	Country
Noni et al. (2020)	n/a	*asahii*	F	Catheter	n/a	n/a	Pacemaker insertion, hypotonia	n/a	n/a	Greece
Basu et al. (2015)	9	*asahii*	M	Blood	920	27	Prematurity, ELBW, RDS, ventilation, LOS	LAMB	Died	India
	8	*asahii*	M	Blood	980	27	Prematurity, ELBW, RDS, CPAP, LOS	LAMB	Died	India
	11	*asahii*	F	Blood	900	30	Prematurity, ELBW, LOS	LAMB	Died	India
Vashishtha et al. (2012)	5	*Trichosporon* spp.	n/a	Blood	2890	Term	MSAF, PNA, gastrointestinal bleeding	AMB	Died	India
	7	*Trichosporon* spp.	n/a	Blood	1550	31	SGA with RDS (HMD), mechanical ventilation	AMB	Died	India
	n/a	*asahii*	n/a	Blood	1235	28	AGA, PNA, PPROM, RDS	AMB	Died	India
	11	*Trichosporon* spp.	n/a	Blood	1720	34	SGA, BOH. PPROM, NEC	AMB	Alive	India
	21	*Trichosporon* spp.	n/a	Blood	1080	29	SGA, PNA, PPROM, mechanical ventilation	AMB	Died	India
	n/a	*asahii*	n/a	Blood	1250	32	SGA, PNA, PPROM, RDS, mechanical ventilation, early onset sepsis	AMB	Died	India
	n/a	*asahii*	n/a	Blood	1200	35	SGA, polycythemia, sepsis	AMB	Died	India
	n/a	*asahii*	n/a	Blood	2400	Term	SGA, PNA, sepsis	AMB	Alive	India
Pereira et al. (2009)	n/a	*asahii*	M	Blood	815	29	RDS, mechanical ventilation	n/a	Died	Brazil
Chagas-Neto et al. (2009)	16	*asahii*	F	Blood	n/a	n/a	Prematurity	AMB	Died	Brazil
	84	*asahii*	M	Blood	n/a	n/a	Premature birth, enterectomy	FLC+AMB	Alive	Brazil
Tellez-Castillo et al. (2008)	n/a	*asahii*	n/a	Endovascular catheter, tube	685	24	Prematurity, sepsis, mechanical ventilation	n/a	Died	Spain
Maheshwari et al. (2004)	26	*asahii*	F	Blood	737	24	RDS, mechanical ventilation, PDA, sepsis	AMB + 5-FC	Alive	USA
Yildiran et al. (2003)	21	*asahii*	F	Blood, urine	1050	27	Prematurity, RDS, LOS	AMB	Alive	Turkey
Salazar et al. (2002)	15	*beigelii*	M	Blood, tracheal aspirate	960	27	RDS, mechanical ventilation, sepsis	AMB	Died	USA
	17	*asahii*	M	Peritoneal fluid, blood, urine	720	24	RDS, mechanical ventilation, PPROM	AMB	Died	USA
Panagopoulou et al. (2002)	6	*asahii*	F	Blood	890	26	RDS, LOS	AMB	Alive	Greece
Sweet et al. (1998)	16	*beigelii*	F	Tracheal aspirate, peritoneal fluid, skin, urine	950	25	RDS, PPROM	LAMB	Alive	United Kingdom
Yoss et al. (1997)	10	*beigelii*	M	Blood, umbilical catheter, tracheal aspirate	530	23	RDS, sepsis, mechanical ventilation	AMB	Died	USA
	10	*beigelii*	F	Urine, tracheal aspirate, umbilical catheter	545	23	RDS, mechanical ventilation, sepsis	AMB	Died	USA

Abbreviations for Table 1. ELBW: Extremely low birth weight, RDS: Respiratory distress syndrome, LOS: Late onset sepsis, CPAP: Continuous positive airway pressure, MSAF: Meconium-stained amniotic fluid, PNA: Perinatal asphyxia, SGA: Small for gestational age, HMD: Hyaline membrane disease, AGA: Appropriate for gestational age, PPROM: Prolonged Premature Rupture of the Membranes, NEC: Necrotizing enterocolitis, BOH: Bad Obstetric History, PDA: Patent Ductus Arteriosus, LAMB: Liposomal Amphotericin B, AMB: Amphotericin B, FLC: Fluconazole, 5-FC: Flucytosine, M: Male, F: Female, n/a: not available, USA: United States of America.

**Table 2:** Characteristics of Invasive *Trichosporon* Infections (ITI) in pediatric patients with malignant and/or hematologic diseases.

Study (Year)	Sex, Age (yrs)	Underlying Disease	ANC<500	Site of Isolation	Antifungal Prophylaxis	Empirical Antifungal Treatment	Treatment	Outcome	Country
Noni et al. (2020)	F, 2.5	Yolk sac tumor	n/a	Blood	n/a	n/a	n/a	n/a	Greece
	M, 14	Relapsed ALL after BMT	n/a	Blood	n/a	n/a	n/a	n/a	Greece
	F, 10	ALL	n/a	Pleural fluid	n/a	n/a	n/a	n/a	Greece
	n/a, 10	Blackfan-Diamond	n/a	Blood, Bronchial secretion	n/a	n/a	n/a	n/a	Greece
Raju et al. (2019)	M, 1	Wilms	YES	Blood	n/a	n/a	VRC	Alive	India
Galligan et al. (2018)	M,18	Relapsed ALL	n/a	Blood, Skin	MCF	AMB, VRC	VRC	Died of ALL	USA
Lee Yuexian et al. (2017)	F, 4	Aplastic anemia	YES	Skin	CAS	LAMB	VRC	Alive	Singapore
Nguyen et al. (2017)	M, 10	High-risk ALL	YES	Lung, heart, kidney, spleen, lymph nodes	MFG	LAMB	VRC	Died	USA
Foster et al. (2016)	M, 10	ALL	YES	Blood, lung	MFG	LAMB	LAMB, VRC	Died	USA
	F, 15	ALL	YES	Skin	MFG	LAMB	LAMB, VRC	Alive	USA
	F, 8	ALL	YES	Skin	MFG	LAMB, VCZ	LAMB, VRC	Alive	USA
Maxfield et al. (2015)	F, 3	ALL	n/a	Blood, Urine, Skin	n/a	VCZ, MCF	LAMB, VRC	Died of other cause	USA
Oh et al. (2015)	M, 3	Mixed ALL/AML	YES	Skin	n/a	MCF	AMB, POS	Alive	USA
Tanyildiz et al. (2015)	M, 2	LCH	YES	Blood	n/a	LAMB	VRC	Alive	Turkey
	F, 12	Secondary AML	YES	Blood	n/a	LAMB	VRC	Alive	Turkey
Karapinar et al. (2014)	F,16	Aplastic anemia	YES	Blood	n/a	CAS	VRC	Died	Turkey
	F, 5	ALL	YES	Blood	n/a	CAS	VRC	Alive	Turkey
Agarwal and Joyce (2014)	n/a, 12	AML	YES	Blood	FLC	n/a	n/a	Died	USA
	n/a, 12	AML	YES	Blood	CAS	n/a	n/a	Died	USA
	n/a, 12	AML	YES	Urine	MFG	n/a	n/a	n/a	USA
	n/a, 12	AML	YES	Urine	VCZ, MFG	n/a	n/a	n/a	USA
	n/a, 13	AML	YES	Blood	VCZ	n/a	n/a	Died	USA
	n/a, 14	AML	YES	Blood	MFG	n/a	n/a	n/a	USA
Parlakay et al. (2013)	M, 16	Ewing	n/a	Blood, Conchae, nose	n/a	LAMB, CAS,	LAMB	Died	Turkey
Kudo et al. (2011)	F, 0.4	AML	YES	Blood	AMB	MFG	VCZ	Alive	Japan
Thibeault et al. (2008)	M, 11	ALL	YES	Blood, liver, urine	n/a	L-AMB	VCZ	Died	Canada
Tsuji et al. (2008)	M,16	ALL	NO	Blood, urine	MFG	VRC	VCZ	Died of other cause	Japan
Hosoki et al. (2008)	M, 18	MDS	n/a	Blood	AMB, ITC	LAMB, ITC	LAMB, ITC	Died of other cause	Japan
Ghiasian et al. (2006)	F, 11	Aplastic anemia	YES	Blood, sputum, oral lesions	n/a	AMB	AMB	Died	Iran
Antachopoulos et al. (2005)	M, 13	ALL	YES	Blood, BAL	AMB	LAMB	LAMB, VCZ	Died of other cause	Greece
Meyer et al. (2002)	n/a, 13	Mixed ALL/AML	YES	Blood, liver	n/a	AMB	ITC	Alive	France
Itoh et al. (1996)	F, 5	ALL	YES	Blood, skin	n/a	n/a	MFG	Died	Japan

Abbreviations for Table 2: AMB: Amphotericin B, LAMB: Liposomal Amphotericin B, AFG: Anidulafungin, CAS: Caspofungin, 5FC: Flucytosine, FLC: Fluconazole, ITC: Itraconazole, MFG: Micafungin, POS: Posaconazole, VRC: Voriconazole, ALL: Acute Lymphoblastic Leukemia, AML: Acute Myeloid Leukemia, n/a: not available, M: Male, F: Female, USA: United States of America, BMT: Bone Marrow Transplantation, LCH: Langerhans Cell Histiocytosis, BAL: Bronchoalveolar Lavage.

**Table 3:** MICs (μg/mL) of antifungal agents for *T. asahii* isolates in studies with pediatric malignant and/or hematologic disorders.

Study (Year)	Species	AMPB	5-FC	Fluconazole	Itraconazole	Voriconazole	Posaconazole	Caspofungin	Micafungin	Anidulafungin
Noni et al. (2020)	Yolk sac	0.125	8	4	0.03	0.01	0.031	8	8	8
	Relapsed ALL after BMT	8	8	6	0.031	0.031	0.25	16	8	8
	ALL	1	8	32	0.06	0.06	0.125	4	8	8
	Blackfan-Diamond	6	8	16	4	0.19	0.031	8	8	8
Raju et al. (2019)	Wilms	sens	n/a	sens	n/a	0.06	n/a	n/a	n/a	n/a
Galligan et al. (2018)	Relapsed ALL	n/a	n/a	n/a	n/a	n/a	n/a	n/a	n/a	n/a
Lee Yuexian et al. (2017)	Aplastic anemia	n/a	n/a	n/a	n/a	n/a	n/a	n/a	n/a	n/a
Nguyen et al. (2017)	High-risk ALL	n/a	n/a	n/a	n/a	≤0.03	n/a	n/a	n/a	n/a
Foster et al. (2016)	ALL	2	n/a	0.5	0.5	≤0.03	0.06	>8	>8	>8
	ALL	1	n/a	4	0.5	0.125	0.25	>8	>8	>8
	ALL	1	n/a	2	0.5	0.06	0.125	≥8	≥8	>8
Maxfield et al. (2015)	ALL	n/a	n/a	n/a	n/a	n/a	n/a	n/a	n/a	n/a
Oh et al. (2015)	Mixed ALL/AML	n/a	n/a	n/a	n/a	n/a	n/a	n/a	n/a	n/a
Tanyildiz et al. (2015)	LCH	1	n/a	0.50	0.125	0.03	0.25	4	n/a	2
	Secondary AML	1	n/a	0.5	0.25	0.06	0.5	3	n/a	3
Karapinar et al. (2014)	Aplastic anemia	1.5	n/a	3	0.032	0.094	n/a	>32	n/a	n/a
	ALL	0.008	n/a	0.023	0.25	0.023	n/a	>32	n/a	n/a
Agarwal and Joyce (2014)	AML	n/a	n/a	n/a	n/a	n/a	n/a	n/a	n/a	n/a
	AML	n/a	n/a	n/a	n/a	n/a	n/a	n/a	n/a	n/a
	AML	n/a	n/a	n/a	n/a	n/a	n/a	n/a	n/a	n/a
	AML	n/a	n/a	n/a	n/a	n/a	n/a	n/a	n/a	n/a
	AML	n/a	n/a	n/a	n/a	n/a	n/a	n/a	n/a	n/a
	AML	n/a	n/a	n/a	n/a	n/a	n/a	n/a	n/a	n/a
Parlakay et al. (2013)	Ewing	n/a	n/a	4	n/a	0.03	n/a	n/a	n/a	n/a
Kudo et al. (2011)	AML	2	>64	4	1	0.125	n/a	n/a	>16	n/a
Thibeault et al. (2008)	ALL	8	n/a	1	n/a	0.03	n/a	>8	n/a	n/a
Tsuji et al. (2008)	ALL	0.5	n/a	1	0.25	0.25	n/a		>16	n/a
Hosoki et al. (2008)	MDS	2	>128	>128	2				≥32	
Ghiasian et al. (2006)	Aplastic anemia	n/a	n/a	n/a	n/a	n/a	n/a	n/a	n/a	n/a
Antachopoulos et al. (2005)	ALL	0.25	n/a	4	0.5	0.125	0.5	n/a	n/a	n/a
Meyer et al. (2002)	Mixed ALL/AML	0.032	n/a	2	2	n/a	n/a	n/a	n/a	n/a
Itoh et al. (1996)	ALL	0.25	n/a	n/a	n/a	n/a	n/a	n/a	n/a	n/a
Median MIC (pediatric studies)		1	8	3.5	0.375	0.06	0.187	8	8	8

Abbreviations for Table 3: AMP: Amphotericin B, 5-FC: Flucytosine, ALL: Acute Lymphoblastic Leukemia, AML: Acute Myeloid Leukemia, n/a: Not available, Sens: Sensitive, MIC: Minimal Inhibitory Concentration.

## Data Availability

Not applicable.
